# Assessing Oncologic and Functional Outcomes of 3D Image-Guided Robotic-Assisted Partial Nephrectomy (3D-IGRAPN): A Prospective Study (UroCCR-186)

**DOI:** 10.3390/cancers17132127

**Published:** 2025-06-25

**Authors:** Alice Pitout, Gaëlle Margue, Federico Rubat Baleuri, Abderrahmane Khaddad, Maxime Pattou, Franck Bladou, Grégoire Robert, Jean-Christophe Bernhard

**Affiliations:** 1 Urology Department, Bordeaux University Hospital, 33000 Bordeaux, France; 2 I.CaRe Bordeaux—BRIC Inserm U1312, 33200 Bordeaux, France; 3 French AFU Cancer Committee Guidelines, 75017 Paris, France

**Keywords:** 3D-IGRAPN, robotic partial nephrectomy, renal carcinoma, 3D model, image-guided surgery, nephron-sparing surgery, trifecta

## Abstract

**Simple Summary:**

Robotic partial nephrectomy is a standard treatment for localized kidney tumors, aiming to preserve renal function while ensuring oncologic control. The use of three-dimensional (3D) image-guided techniques allows surgeons to plan procedures with enhanced anatomical precision based on patient-specific imaging. In this study, we evaluated the outcomes of over 500 cases using 3D image-guided robotic-assisted partial nephrectomy (3D-IGRAPN) performed at a high-volume center. We found that this technique is associated with low complication rates, good preservation of kidney function, and cancer control—even in anatomically complex or high-risk cases. These results support the broader integration of 3D planning into minimally invasive kidney surgery and suggest its potential to expand nephron-sparing indications.

**Abstract:**

Background/Objectives: Three-dimensional (3D) image-guided robotic-assisted partial nephrectomy (3D-IGRAPN) integrates patient-specific anatomical models to optimize surgical planning and intraoperative guidance in the management of renal tumors. This study aimed to assess medium-term functional and oncologic outcomes of 3D-IGRAPN in a large, prospective cohort. Methods: All consecutive patients undergoing 3D-IGRAPN between January 2016 and March 2023 at a tertiary referral center were prospectively included in the UroCCR database (NCT03293563). Patient-specific 3D models were generated from preoperative CT scans and used intraoperatively. The primary endpoint was trifecta achievement, defined as an absence of major complications (Clavien–Dindo ≥ 3), negative surgical margins for malignant tumors, and ≥90% preservation of baseline renal function at 3 months. Secondary endpoints included functional outcomes, complication rates, local recurrence, and metastasis rates, as well as cancer-specific and overall survivals. Results: Among 568 patients (586 surgeries), the trifecta was achieved in 55.2% of evaluable malignant cases. Severe complications occurred in 33 cases (5.6%), and positive surgical margins were reported in 27 cases (5.1%) out of 528 surgeries involving malignant lesions. Renal function was preserved in 59.9% of patients at 3 months. At a mean follow-up of 31.5 months, recurrence and metastasis rates were 7.4% and 8.6%, respectively. Cancer-specific and overall survival at follow-up were 96.5% and 89%. Conclusions: 3D-IGRAPN demonstrates favorable functional and oncologic outcomes, even in complex tumors. These results support the integration of 3D modeling as a standard tool in image-guided nephron-sparing surgery.

## 1. Introduction

Partial nephrectomy is the established standard of care for treating localized renal tumors, providing an optimal balance between tumor resection and preservation of renal function when technically feasible [1]. This approach aligns with the fundamental goals of renal oncological surgery, commonly referred to as the “trifecta”—achieving nephron-sparing and ensuring oncological safety while minimizing warm ischemia and postoperative complications [2].

In recent years, minimally invasive robotic surgery has gained prominence, offering distinct advantages that support these objectives. Compared to laparoscopic techniques, robotic-assisted methods reduce ischemia time and enhance postoperative renal clearance [3], alongside offering less bleeding or post-operative complications and shorter hospital stay than the traditional open approach [4]. Furthermore, the robotic approach increases the feasibility of complex partial nephrectomy, resulting in lower conversion rates to radical nephrectomy compared to the laparoscopic approach [5]. With technological advancements, new tools, such as indocyanine green, ultrasound, and pre-operative 3D modeling are being explored to further enhance surgical outcomes [6]. Among these, 3D image-guided robotic-assisted partial nephrectomy (3D-IGRAPN) emerges as a transformative technique. This method involves creating a digital twin of the kidney from preoperative CT scans, facilitating strategic surgical planning and a comprehensive understanding of kidney anatomy in three dimensions during the surgery.

Over the past eight years, our center has extensively employed the 3D-IGRAPN technique, previously demonstrating its superiority in achieving trifecta outcomes compared to traditional approaches in a four-year comparative analysis [7]. The current study aims to evaluate the medium-term outcomes of the 3D-IGRAPN technique, offering further evidence of its efficacy and potential role in advancing renal surgery.

## 2. Materials and Methods

We prospectively included all patients managed with 3D-IGRAPN at our center between January 2016 and March 2023 using Da Vinci Si or Xi platforms [8,9]. Each patient provided informed consent and was subsequently enrolled in the UroCCR database, a comprehensive national multicenter registry that prospectively captures clinical, biological, and radiological data on kidney cancer (NCT03293563) [10]. This study received approval from the Institutional Review Board (CNIL DR-2022-091; CPP DC-2012/108).

**Figure 1:**
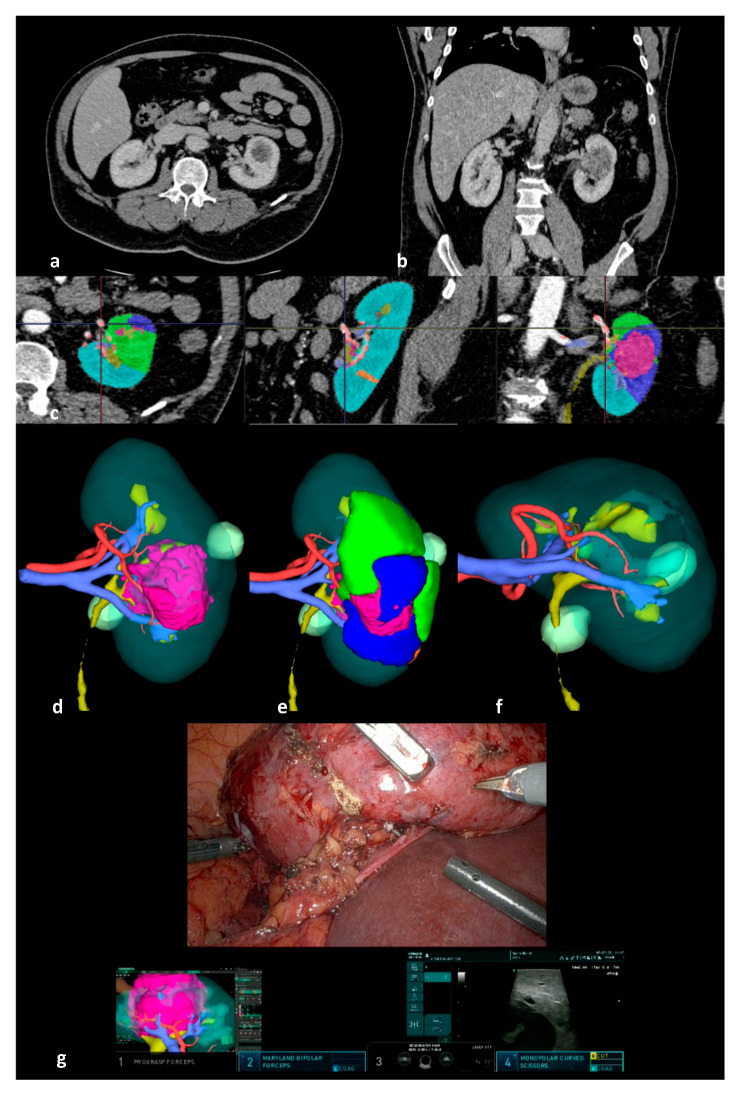
Pre- and intraoperative visualization during a 3D image-guided robot-assisted partial nephrectomy (3D-IGRAPN). (**a**) Axial CT scan; (**b**) coronal CT scan; (**c**) CT scan segmentation showing the renal parenchyma in light blue, the tumor in pink, and simulated ischemic territories according to clamping points in green and dark blue; (**d**) 3D model of the tumor-bearing kidney; (**e**) simulation of ischemic territories on the 3D model; (**f**) simulation of the tumor resection bed; (**g**) intraoperative console view with Tile Pro display during 3D-IGRAPN.

Eligible participants were adult patients presenting with renal tumors managed using 3D-IGRAPN. The pre-operative 3D models were created using Fujifilm’s Synapse software (version 4.4, Tokyo, Japan) from a pre-operative 4-phase CT scan [11]. The segmentation was performed by a urologist or a trained resident, detailing a 3D representation of the renal parenchyma, tumor, excretory system, vasculature, and potential cysts (Figure 1). The choice to employ the 3D-IGRAPN technique was determined at the surgeon’s discretion.

Demographic, clinical, surgical, and post-operative follow-up data were prospectively recorded for each patient.

The primary endpoint of this study was the achievement of the trifecta, defined as follows [2]: maintenance of post-operative renal clearance (eGFR CKD-EPI) at or above 90% of pre-operative level at the first follow-up (3 months), absence of severe peri-operative complications (classified as Clavien–Dindo grade ≥ 3) occurring intra-operatively or within three months postoperatively, and negative surgical margins for malignant tumors.

Secondary endpoints encompassed functional and oncological outcomes. Functional outcomes included measurements of serum creatinine (µmol/L) and renal clearance (eGFR CDK-EPI) at multiple time points: post-operative day 1, at discharge, at 3, 6, 9, 12, and 24 months, and at the last follow-up. Additional functional assessments involved the incidence of acute kidney injury (AKI) (defined by a 25% reduction in eGFR at discharge), changes in chronic kidney disease classification (worsening by at least one stage), rates of serious intra- and post-operative complications, rates of ambulatory surgery, and length of hospital stay. Oncological outcomes assessed were local recurrence and distant metastasis rates, cancer-specific survival, and overall survival. In patients with multiple tumors, the largest tumor was used for outcome evaluation.

Statistical analyses were conducted utilizing R version 4.4.1 (R Core Team, 2024, Vienna, Austria). Descriptive statistics were employed to summarize the data, with qualitative variables presented as frequencies and percentages and quantitative variables as means and standard deviations. Given the exploratory nature of this study, no formal hypothesis testing or comparative analyses were performed.

Survival analyses were executed using the Kaplan–Meier method to estimate survival probabilities and evaluate time-to-event outcomes. Survival curves were generated to visually represent these estimates, clearly indicating censored data to account for participants lost to follow-up or who had not experienced the event by the conclusion of the study.

In addition to the primary trifecta definition (negative surgical margins, no major complications, and ≥90% renal function preservation at 3 months, as proposed by Hung et al. [2]), we performed a complementary analysis using an alternative trifecta definition proposed by Brassetti et al. [12]. This definition includes negative surgical margins, no major complications (Clavien–Dindo ≥ 3), and a post-operative eGFR reduction ≤ 30%. This secondary analysis was intended to facilitate comparison with other published series using this alternative trifecta definition.

## 3. Results

During the study period, among a total number of 979 RAPN, 586 (59.9%) 3D-IGRAPN surgeries were performed on 568 patients. The mean follow-up duration was 31.5 ± 21.8 months. Detailed population characteristics are outlined in Table 1.

The cohort predominantly consisted of male patients (70.1%), with a mean age of 59.9 ± 13.8 years. Indication for nephron-sparing surgery (NSS) was imperative in 17.7% of cases, and 8.4% of surgeries were performed for multiple tumors.

The mean tumor size was 4.9 cm ± 2.4, and the mean RENAL score was 8.4 ± 1.7 with a large majority of cases classified as complex or highly complex tumors.

Regarding the surgical technique, 60% of the procedures were performed using an off-clamp approach, while a sutureless technique was employed in 15% of cases.

The trifecta was evaluated in a subset of 489 patients with malignant tumors and documented eGFR, as surgical margin considerations are not applicable to benign lesions. Among these patients, the 3D-IGRAPN procedure satisfied the trifecta in 270 cases, representing 55.2% of the cohort. When stratified by tumor complexity, trifecta achievement was 62.0% for low, 58.8% for moderate, and 46.6% for high complexity lesions.

When evaluating the components of the trifecta independently across the full cohort of 586 surgeries, severe perioperative complications occurred in 33 cases (5.6%), and positive surgical margins were reported in 27 cases (5.1%) among the 528 procedures performed for malignant lesions. Furthermore, among the 538 patients with available eGFR at 3 months postoperatively, renal function was preserved above 90% of the baseline in 322 patients (59.9%).

Applying the alternative trifecta definition proposed by Brassetti et al. [12], we found that 72.1% of patients with malignant tumors achieved trifecta.

Acute kidney injury (AKI) was identified in 123 patients, representing 21% of the cohort. At the initial follow-up at 3 months, patients exhibited an average decline in glomerular filtration rate (GFR) of 6.4 ± 26.69 points from their pre-operative baselines. As a result, 24% of patients experienced a progression of at least one stage of chronic kidney disease (CKD). These results remained stable over time at 6, 9, 12, and 24 months (Table 2).

By the final follow-up, the average reduction in GFR increased to 8.9 ± 13.2 points, leading to CKD upstaging by at least one class in 32.4% of patients. Among patients with a CKD > 90 mL/min pre-operatively, 13.2% had a CKD < 90 mL/min at 3 months.

**Figure 2:**
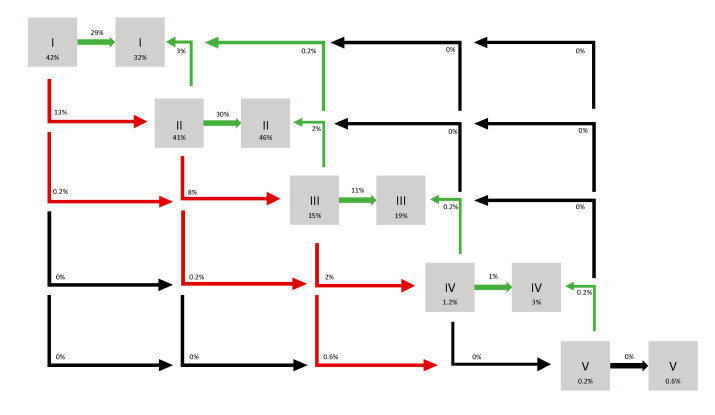
Evolution of CKD stages at 3 months. I: eGFR > 90 mL/min; II: eGFR between 60 and 90 mL/min; III: eGFR between 30 and 60 mL/min; IV: eGFR between 15 and 30 mL/min; V: eGFR < 15 mL/min.

Figure 2 provides a visual representation of CKD stage evolution at first follow-up, indicating that 76.4% of patients maintained stable or improved renal function, with 78% retaining a GFR of more than 60 mL/min.

In terms of hospitalization metrics, the average length of stay was 2 ± 4.4 days, with 9.2% of the cohort undergoing outpatient surgery (less than 12 h in hospital).

The mean follow-up was 31.5 ± 21.8 months. During this time, among patients with malignant disease, we observed locoregional recurrence in 42 patients (7.4%) and metastatic progression in 49 patients (8.6%).

**Figure 3:**
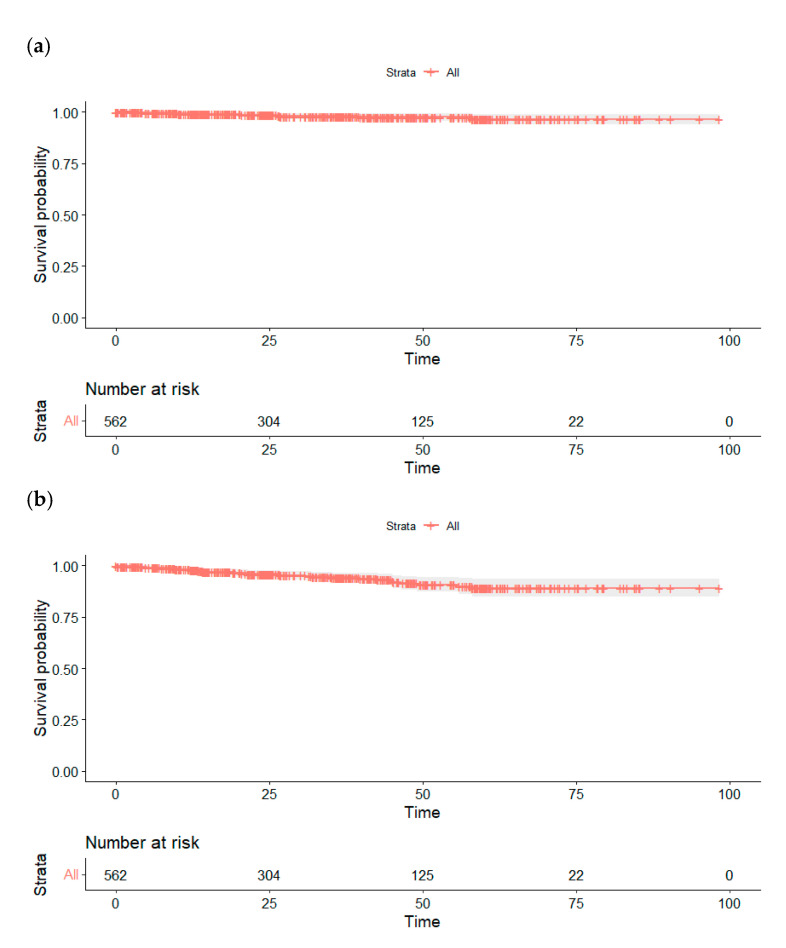
Survival results, Kaplan–Meier analysis. (**a**) Cancer-specific survival; (**b**) overall survival.

Kaplan–Meier survival curves are presented in Figure 3, reporting 5-year cancer-specific and overall survival rates of 96.5%, and 89.0%, respectively.

## 4. Discussion

Over the past eight years, the 3D-IGRAPN technique has become a key element of our surgical strategy. In this study, we analyzed all procedures performed during this period, predominantly involving complex renal tumors—84% with intermediate or high RENAL scores. Additionally, 17.7% of surgeries were performed for imperative indications, and multiple tumorectomies were carried out in 8.4% of cases. Off-clamp surgery was achieved in 60% of patients, and a sutureless technique was used in 15%, underscoring the precision afforded by this image-guided approach.

Surgical morbidity was limited, with moderate blood loss, a low transfusion rate—even in off-clamp procedures—and a serious complication rate of only 5.6%. More than 75% of patients were discharged within 24 h, and renal function remained stable or improved at first follow-up, including in patients with pre-existing renal insufficiency. Importantly, outcomes were particularly encouraging in patients with preoperative stage III to V CKD, in whom the rate of CKD upstaging at first follow-up was low, reflecting the renal preservation potential of 3D-IGRAPN even in vulnerable populations. These results suggest that 3D-IGRAPN may facilitate safe resection in complex cases.

This favorable profile aligns with a trifecta achievement rate of 55.2%. We previously reported similar outcomes in our 2021 study involving 230 patients, showing a similar trifecta rate, a major complication rate of 4.3%, and a minimal eGFR variation of −7% at early follow-up [7]. That cohort also showed low recurrence and metastasis rates (2.6% and 3.9%, respectively), with a median follow-up of 11.7 months. The present analysis, involving a cohort twice as large and a longer mean follow-up of 31.5 months, reinforces these initial findings and supports the technique’s reproducibility across different surgeons in a real-world context. While our primary endpoint was based on the trifecta definition by Hung et al. [2], we also explored the alternative definition by Brassetti et al. [12]. This complementary analysis confirmed a high trifecta rate (72.1%), demonstrating the robustness of our results regardless of the criteria used.

Importantly, trifecta outcomes were consistent across the complexity spectrum, though a progressive decrease was observed with increasing tumor complexity: 62.0% in low-complexity cases, 58.8% in moderate-complexity, and 46.6% in high-complexity tumors. These results suggest that while 3D-IGRAPN maintains a strong performance even in challenging cases, tumor complexity remains a limiting factor for achieving all trifecta criteria—particularly in terms of ischemia-free dissection and complication avoidance. Nonetheless, the relatively high trifecta rate in high-complexity tumors underscores the value of preoperative 3D planning in supporting surgical precision and preserving functional outcomes. Totally endophytic tumors present unique challenges during partial nephrectomy due to their limited intraoperative visibility and complex anatomical relationships. In our experience, 3D models enable precise preoperative visualization of tumor position relative to surrounding vasculature and the collecting system. This facilitates safe and accurate resection, potentially reducing the risk of positive surgical margins and injury to critical structures.

Our results are consistent with prior literature demonstrating the advantages of RAPN over open and laparoscopic approaches. Several studies have shown higher trifecta success, fewer complications, and faster recovery with robotic platforms [13,14,15,16]. In our series, robotic assistance and 3D guidance were associated with low morbidity, short hospital stays, and the ability to safely perform off-clamp and sutureless procedures—even in complex anatomical settings—thus expanding the scope of conservative surgery in renal oncology.

The use of 3D virtual models has further enhanced these benefits. Grosso et al. [17] demonstrated that, in high-complexity tumors (PADUA ≥10), the addition of a 3D model to RAPN improved renal function at one year, beyond what could be achieved by selective clamping and enucleation alone. Our findings on CKD progression are consistent with those reported in other large cohorts of partial nephrectomy, including the recent study by Flammia et al. [18], which also included patients undergoing off-clamp, on-clamp, and selective clamping approaches with comparable tumor complexity. In their cohort, the authors reported CKD upstaging rates at one year, similar to those observed in our series. This concordance reinforces the impact of tumor complexity and clamping strategies on postoperative renal function. Our data, along with theirs, highlight the importance of meticulous preoperative planning, including the use of 3D modeling, to optimize clamping strategy and preserve renal function, even in challenging anatomical scenarios. Multiple studies [19,20,21,22] have similarly reported that 3D model-guided RAPN results in shorter hospital stays, reduced blood loss, less renal function impairment, and higher trifecta success. These findings have been confirmed in meta-analyses [23,24], which emphasize reduced ischemia time, more precise clamping, and better preservation of vascular territories. Beyond clinical impact, 3D models have also proven useful for surgical planning, resident training, and improved patient understanding [25]. In our practice, 3D models were not only reviewed preoperatively but also integrated intraoperatively in the robotic console to guide real-time surgical decision making. This facilitated the planning of clamping strategies and sutureless resection in selected cases. However, it was not planned in the design of our study to systematically evaluate how the 3D model influenced each intraoperative choice. Future prospective studies will be needed to better assess this impact on surgical strategy and outcomes.

However, significant heterogeneity persists in the literature regarding model types (digital vs. printed), surgical approaches (laparoscopic vs. robotic), and outcome measures. Many studies involve small cohorts or mixed populations. In particular, the definition of trifecta varies widely—some omit it altogether, while others report up to a 30% variation in success rates depending on the criteria used [19,26]. This lack of standardization makes direct comparisons difficult and reinforces the need for consensus in outcome reporting.

To our knowledge, this is the largest single-center series evaluating 3D-IGRAPN. Data were collected prospectively via the UroCCR registry [10], reducing selection bias and reflecting real-life surgical practice with varying levels of operator experience. Nonetheless, our study is limited by the absence of a control group and a potential selection bias, as 3D-IGRAPN was preferentially used in more complex or challenging cases. Given the widespread adoption of the technique in our center, creating a matched comparison group was not feasible. We acknowledge that the lack of a comparator group is a limitation of our study and may introduce selection bias. As 3D-IGRAPN became the standard approach in our center—including for lower-complexity tumors—it became impossible to define a historical control group with similar characteristics and obtain a matched cohort for comparison. On the other hand, although our study design does not allow for a formal analysis of temporal trends in outcomes related to increasing experience with 3D-IGRAPN, we acknowledge that both surgeon experience and iterative improvements in 3D model utilization could have contributed to enhanced perioperative outcomes over time. This learning effect is an important consideration, and ongoing multicenter randomized studies are expected to provide more definitive insights into the impact of 3D models on surgical planning and outcomes.

Future prospective randomized studies, including the ongoing ACCURATE trial (UroCCR 99) [27], are needed to more rigorously assess the added value of 3D models in nephron-sparing surgery. Conducted within the Digital Urology 3D program, the ACCURATE trial (UroCCR 99) compares outcomes between 3D-IGRAPN and standard RAPN without 3D modeling across 14 French centers, aiming to provide high-level evidence for the technique’s added value.

Looking ahead, the integration of augmented reality (AR) into robotic surgery represents a promising next step. By overlaying real-time anatomical information directly into the surgical field, AR may further improve precision, reduce operative time, and extend the feasibility of minimally invasive approaches [28,29]. Combined with 3D planning, AR could help establish a new standard in image-enhanced surgery for renal tumors.

## 5. Conclusions

This study highlights the efficacy of 3D-IGRAPN in managing localized renal tumors, with high trifecta achievement and excellent outcomes in complex nephron-sparing surgeries. The integration of 3D modeling may enhance surgical precision, reducing operative time, blood loss, and warm ischemia. These results from the largest-to-date 3D-IGRAPN cohort position the technique as a promising advancement in renal oncology surgery. Further multicentric comparative prospective studies are needed to validate these findings and refine trifecta criteria as a standard benchmark in nephron-sparing procedures.

## Figures and Tables

**Table 1:** Characteristics of the cohort. In cases involving multiple tumors, the characteristics of the largest tumor were used for data presentation. NSS: nephron-sparing surgery, SD: standard deviation.

**Patients Characteristics**	**n = 568**
Age at surgery (years), mean (SD)	59.9 (13.8)
Sex, n (%)	
Male	398 (70.1)
Female	170 (29.9)
cT stage	
1a	219 (38.6)
1b	233 (41)
2a	61 (10.7)
2b	17 (3.0)
3a	14 (2.5)
NR	24 (4.2)
**Surgery and Tumor Characteristics**	**n = 586**
Preoperative (eGFR mL/min), mean (SD)	85.6 (22.5)
NSS Indication, n (%)	
Elective	473 (80.7)
Imperative	104 (17.7)
Relative	45 (7.7)
Multiple tumorectomies, n (%)	49 (8.4)
Tumor size (cm), mean (SD)	4.9 (2.4)
RENAL score, mean (SD)	8.4 (1.7)
Complexity according to RENAL score, n (%)	
Low	93 (15.9)
Moderate	298 (50.8)
High	195 (33.3)
Clamping type, n (%)	
Main artery	80 (13.7)
Superselective	151 (25.8)
Off-clamp	353 (60.2)
Warm ischemia time in case of main artery clamping (min), mean (SD)	22.0 (14.5)
Renorrhaphy, n (%)	
None (sutureless)	85 (14.5)
Parenchymal suture (single layer)	294 (50.2)
Capsular + parenchymal suture (double layer)	207 (35.3)
Blood loss (mL), mean (SD)	322.3 (360.4)
Intra-operative transfusion rate, n (%)	14 (2.4)
Conversion to open surgery, n (%)	2 (0.3)
Conversion to radical nephrectomy, n (%)	2 (0.3)
Operative time (min), mean (SD)	203.2 (78.1)
Malignant lesion, n (%)	528 (90.1)
Histological subtype, n (%)	
Clear cell carcinoma	369 (63.0)
Papillary	62 (10.6)
Chromophobe	54 (9.2)
Other malignant tumors	43 (7.3)
Oncocytoma	44 (7.5)
Benign cyst	9 (1.5)
Angiomylipoma	5 (0.9)
pT stage	
1a	231 (39.4)
1b	119 (20.3)
2a	22 (3.8)
2b	3 (0.5)
3a	168 (28.7)
3b	1 (0.2)
NR	42 (7.2)
Length of hospital stay (days), mean (SD)	2.1 (4.4)
Hospital stay, n (%)	
Ambulatory (less than 12 h)	54 (9.2)
Discharge at day 1	350 (59.7)

**Table 2:** Functional outcomes.

	Preoperative	Day 1	3 Months	6 Months	9 Months	12 Months	24 Months	Last Follow-Up
**GFR _1_** **according to CKD-EPI formula (mL/min), mean ± SD _2_**	85.6 ± 22.5	60.9 ± 25.74	77.3 ± 24.01	75.85 ± 25.83	72.14 ± 22.05	74.18 ± 23.96	73.39 ± 26.44	76.4 ± 22.9
**Change in GFR _1_** **compared to preoperative (mL/min), mean ± SD _2_**	—	−22.55 ± 16.76	−6.4 ± 26.69	−10.29 ± 17.86	−7.62 ± 12.74	−6.85 ± 14.43	−8.43 ± 16.07	−8.9 ± 13.2

_1_ GFR, glomerular filtration rate; _2_ SD, standard deviation.

## Data Availability

The datasets presented in this article are not readily available because the data are part of an ongoing study.

## Abbreviations

The following abbreviations are used in this manuscript:

3D	Three-Dimensional
3D-IGRAPN	3D Image-Guided Robotic-Assisted Partial Nephrectomy
eGFR	Estimated Glomerular Filtration Rate
NSS	Nephron-Sparing Surgery
SD	Standard Deviation
AKI	Acute Kidney Injury
